# Peptide-Based Vaccines in Clinical Phases and New Potential Therapeutic Targets as a New Approach for Breast Cancer: A Review

**DOI:** 10.3390/vaccines10081249

**Published:** 2022-08-03

**Authors:** María Lilia Nicolás-Morales, Arianna Luisa-Sanjuan, Mayralina Gutiérrez-Torres, Amalia Vences-Velázquez, Carlos Ortuño-Pineda, Mónica Espinoza-Rojo, Napoleón Navarro-Tito, Karen Cortés-Sarabia

**Affiliations:** 1 Laboratorio de Inmunobiología y Diagnóstico Molecular, Facultad de Ciencias Químico Biológicas, Universidad Autónoma de Guerrero, Chilpancingo de los Bravo 39090, Mexico; moraleslilia009@gmail.com (M.L.N.-M.); ari_1.8@hotmail.com (A.L.-S.); mayralinagutierrez@hotmail.com (M.G.-T.); ameliavences.v@uagro.mx (A.V.-V.); 2 Laboratorio de Ácidos Nucleicos y Proteínas, Facultad de Ciencias Químico Biológicas, Universidad Autónoma de Guerrero, Chilpancingo de los Bravo 39090, Mexico; ortunoc@outlook.com; 3 Laboratorio de Biología Molecular y Genómica, Facultad de Ciencias Químico Biológicas, Universidad Autónoma de Guerrero, Chilpancingo de los Bravo 39090, Mexico; moniespinoza@yahoo.com; 4 Laboratorio de Biología Celular del Cáncer, Universidad Autónoma de Guerrero, Chilpancingo de los Bravo 39090, Mexico; nnavarro@uagro.mx

**Keywords:** peptide-based vaccines, breast cancer, therapeutic, tumor-associated antigens

## Abstract

Breast cancer is the leading cause of death in women from 20 to 59 years old. The conventional treatment includes surgery, chemotherapy, hormonal therapy, and immunotherapy. This immunotherapy is based on administering monoclonal therapeutic antibodies (passive) or vaccines (active) with therapeutic purposes. Several types of vaccines could be used as potential treatments for cancer, including whole-cell, DNA, RNA, and peptide-based vaccines. Peptides used to develop vaccines are derived from tumor-associated antigens or tumor-specific antigens, such as HER-2, MUC1, ErbB2, CEA, FRα, MAGE A1, A3, and A10, NY-ESO-1, among others. Peptide-based vaccines provide some advantages, such as low cost, purity of the antigen, and the induction of humoral and cellular immune response. In this review, we explore the different types of vaccines against breast cancer with a specific focus on the description of peptide-based vaccines, their composition, immune response induction, and the description of new potential therapeutic targets.

## 1. Introduction

Breast cancer is the most diagnosed cancer type and the leading cause of death for cancer in women in developing countries [1]. The conventional treatment includes surgery, radiotherapy, chemotherapy, hormonal therapy, and immunotherapy, although this last was not so relevant until the use of checkpoint inhibitors (anti-CTLA4 and anti-PD-L1) [2]. The objective of immunotherapy is the attack and destruction of cancer cells and includes monoclonal antibodies, chimeric antigen receptor T cell (CAR-T) therapy, and vaccines. Vaccines are based on the capacity of the immune system to identify and differentiate between normal antigens and overexpressed antigens in cancer cells and the generation of immunological memory [3].

Tumor antigens are classified as tumor-associated antigens (TAAs) and tumor-specific antigens (TSA). TAAs are described as overexpressed proteins in cancer cells, and TSAs are proteins expressed exclusively in cancer cells, without expression in normal cells and tissues [4]. TAA and TSA are the primary targets for designing vaccines for cancer. In recent years, several vaccines have been developed based on nucleic acids (DNA and RNA), whole cells, and peptide-based or subunits, among others [5]. One of the most studied TAAs is HER2 (human epidermal growth factor receptor 2); its efficacy as a therapeutic target in breast cancer has been approved, and several therapies and vaccines have been developed [6]. Recently, new TAAs were proposed for the formulation of peptide-based vaccines, including MUC-1 (mucin-1), FRα (folate receptor alpha), members of the MAGE A family (melanoma-associated antigen), and EGFR (epidermal growth factor receptor) [7]. The selected peptide will be inoculated, processed, and presented by immune cells to stimulate responses that promote the destruction of cancer cells expressing the target antigen [8]. However, in addition to all the new proposed therapies, selecting new tumor antigens that could be used as a new therapeutic target is challenging and remains under investigation. Therefore, this review describes the types of therapeutic vaccines against breast cancer and their mechanism of action; specifically, we focus on the description of peptide-based vaccines and new potential therapeutic targets.

## 2. Breast Cancer

Breast cancer (BC) is the main type of cancer in women and the second most frequent cancer worldwide [9]. A total of 1.38 million new cases are reported annually, and it constitutes the leading cause of death in women from 20 to 59 years, with an estimated record of 627,000 cases in 2019, according to reports by the World Health Organization (WHO) [10]. The main etiology of BC remains unknown; however, several factors that contribute to its development have been described and classified as genetics and non-genetics [11]. Epidemiological studies revealed that more than 70% of deaths of BC occur in women aged 40 to 60 years [12], and this risk increases with family background and mutations in the genes *BCRA1* and *2*, *TP53*, *PTEN*, *STK11*, *CDH1*, *PALB2*, *CHECK2*, *ATM*, *NBN,* and *NF1* [13,14]. Reproductive factors, such as menopause, the early onset of menstruation, and pregnancy, are associated with high levels of estrogens and steroid (progesterone and androgens) and non-steroid (insulin) hormones. The hormonal factor has been considered the main factor for the development of BC, in association with activities that involve lifestyle, including alcohol consumption, smoking habit, high-fat diet, and lack of exercise, which are related to the development of obesity and diabetes [15,16].

### 2.1. Types of Breast Cancer

BC could be classified according to the positive expression of HER2 and hormonal receptors for estrogens (ER) and progesterone (PR) as ER/PR-positive, ER/PR-negative, and HER2. The negative expression of ER, PR, and HER2 give place to the “triple-negative” subtype, the most aggressive and with the worst prognostic type of BC [1]. Considering the classification mentioned above, several molecular intrinsic subtypes of BC have been described: luminal A, luminal B, basal, with HER2 overexpression and low claudine [17,18]. The expression of these proteins is associated with the development of carcinomas and sarcomas, of which ductal carcinoma in situ, invasive lobular, and ductal are the most common [19].

The early diagnosis of BC is performed using screening methods, and adequate treatment reduces the mortality rate; the most common clinical evaluations for early detection are mammography, clinical breast test, and self-examination [20]. After a positive diagnosis of BC, several tests need to be performed to determine the stage of the disease [21]. The TNM (T: tumor, N: nodules, and M: metastasis) is the best staging method for neoplasias and stratifies the cancer stage considering the tumor size and type. It was developed by the AJCC (American Joint Committee on Cancer) in collaboration with the UICC (Union for International Cancer Control) [22]. Stage I is described as an invasive carcinoma that has not affected the lymph nodes; this usually occurs in stage II. In contrast, stage III is generally divided into three categories (A, B, and C) with differences in the number of affected lymph nodes. Finally, stage IV describes the dissemination of the tumor to other organs, such as bones, liver, lungs, and brain [23,24].

### 2.2. Treatment of Breast Cancer

After evaluating TNM, there are two treatments: localized surgery and radiotherapy and systemic treatments, including chemotherapy and immunotherapy [25]. Chemotherapy is based on administering one or two drugs that interfere with DNA synthesis and stop tumor growth and cell division. This procedure is efficient but unspecific, evoking damage in healthy tissues and side effects that can contribute to the high mortality rates in patients [26,27]. On the other hand, immunotherapy is defined as any treatment focused on the reduction of the tumor load and the generation of immunological memory [28]. Taking advantage of the comprehension of the innate and adaptive immune response mechanisms involved in the elimination of tumors, new types of passive and active immunotherapies have been developed based on the administration of monoclonal antibodies (passive), the use of immune checkpoint inhibitors, and vaccines (active), for the treatment or prevention of cancer [29,30].

## 3. Vaccines

According to the WHO, vaccines are preparations intended to generate immunological memory against a disease. In the early years, vaccines were produced empirically without knowledge about how the immune system works. To date, the main types of vaccines (live attenuated and inactivated) are based on the experiments performed by Jenner and Pasteur [31,32]. Immune response induction is challenging, and several factors are involved: gender, age, ethnicity, number of doses, genetic factors, and the state of the immune system [33]. Every vaccine must be safe, with defined components, including the purified antigen in suspension or combination with preservative solutions, as well as one or more adjuvants [34,35].

In recent years, the application of novel technologies and the comprehension of immune response mechanisms have allowed the development of vaccines against several diseases. Several methods for the design of vaccines have been reported based on the function of the microorganism as attenuated, inactivated, subunit, and genetic material, that contains the instructions for the production of specific proteins but does not cause the disease [36]. With the development and application of vaccines, several infectious diseases have been eradicated in the last decades. The study of pathogens or the identification of domains and protein subunits derived from specific antigens has laid the foundations for the development of new types of vaccines [37]. Vaccines against cancer provide a new challenge due to their therapeutic approach to controlling the disease. Most of them are found in the clinical phase, and those authorized for their application have shown positive results in different cancers [38,39].

### 3.1. Vaccines against Cancer

Vaccines against cancer are designed to induce an adaptive immune response against the administered antigen. Unlike an infectious disease, the therapeutic immunization to a chronic illness, such as cancer, has several variables [40]. Most cancer vaccines use target molecules named tumor-associated antigens (TAA), described as proteins overexpressed in any type of cancer. TAAs are classified into five categories: mutated antigens expressed exclusively in the tumors, normal antigens overexpressed, oncofetal antigens, differentiation antigens, and cancer/testis antigens [41].

The new advances in the development of vaccines for breast cancer have incorporated different techniques for their design as the fusion of cancer cells with dendritic cells (DC), using the capacity of DC to present antigens [42]. Also, DNA vaccines based on purified antigens imply in vivo transfection of complementary DNA (cDNA) for the production, processing, and presentation of the antigen [40]. All the types of vaccines mentioned above possess their own advantages; however, the less complicated formulation is the administration of peptides derived from TAA mixed with adjuvants. This type of vaccine is more specific and directed without being toxic to the host [43]. Vaccines for cancer are commonly used in therapy, and the best result is observed when they are combined with other types of therapies [44,45,46].

### 3.2. Types of Vaccines against Breast Cancer

The types of cancer vaccines variate according to the route of administration of the TAA as proteins, peptides, whole cells, and nucleic acid [47]. To develop effective vaccines against breast cancer, it is necessary to identify the antigen of interest. Some examples include HER2, MUC-1, and carbohydrate antigens, which have been used in different strategies to amplify immune responses associated with the activation of T cells and antibody production [48]. Vaccines based on the use of proteins or peptides promote the immune response to one or more peptides associated with one specific antigen [49], unlike whole-cell vaccines that contain a higher antigen load with different TAA and a broader immune response [50]. Nucleic acid-based vaccines seek the expression of proteins similar to the target protein of interest. Also, multiple targets could be added to enhance the immune response [51].

#### 3.2.1. Whole-Cell Vaccines

These types of vaccines arise from the idea of taking advantage of the quantity of the antigens associated with the cancer cells, which allows a higher immune response due to their capacity to process and present multiple TAA [42]. This occurs through antigen coupling to the MHC (major histocompatibility complex) class I and II, the recycling of preformed MHC-peptides complex shed by cancer cells, and the peptidic transference of TAA processes by exogenous cells to DC [52].

**Figure 1:**
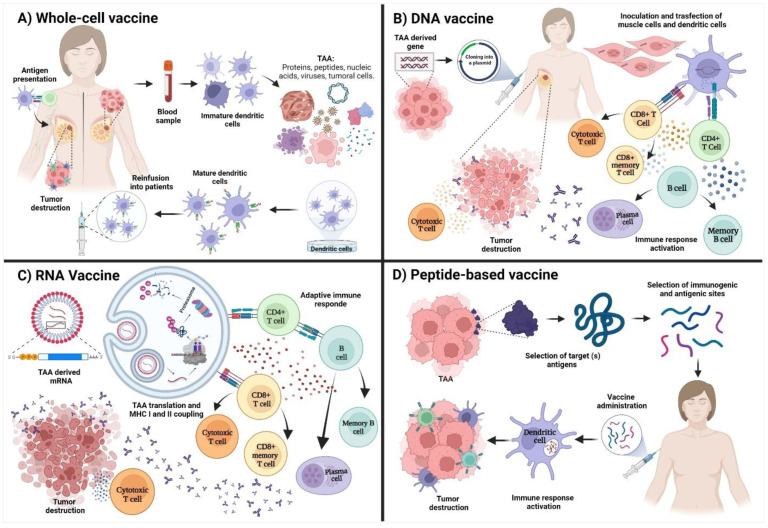
Types and proposed mechanisms of action in cancer vaccines. (**A**) Whole-cell vaccines are based on extracting dendritic cells from the patients. Afterward, they are pulsed with an antigenic load of proteins, peptides, nucleic acids, whole-cell lysates or cancer cells. After the maturation of dendritic cells, they are reinfused into the patient for the induction of an adaptive immune response associated with the destruction of the tumor. (**B**) DNA vaccines are based on amplifying the selected TAA and its cloning into a vector for transfection of muscle cells and dendritic cells that will promote antigen presentation and the activation of T and B cells. (**C**) RNA vaccines are based on the internalization of an mRNA that encodes for a TAA. The mRNA will be translated and processed by the proteasome. The peptides will be coupled to MHC molecules to start the adaptive immune response. (**D**) Peptide-based vaccines are based on the selection of one or multiple TAA and the later selection of peptides using bioinformatic tools as IEDB (http://tools.iedb.org/main/ accessed on 30 July 2022) for the selection of peptides with immunogenic and antigenic characteristics to activate the adaptive immune response. Image created in Biorender (https://biorender.com/ accessed on 2 July 2022).

Using this type of design, Chopra et al., in 2006 [53], formulated a vaccine based on the use of fibroblast transfected with DNA derived from the cell line EO771. The cells were inoculated into CH3/HeJ and C57BL/6J mice in the presence of tumors. Results provided evidence about the effectiveness of the vaccine for antitumor immune response induction associated with the activation of specific T cells against BC. The formulation extended the lifespan of all the animals included in the study. Results improved if the vaccine was applied in combination with paclitaxel, a drug commonly used in the therapy against BC [54]. Another interesting formulation is the use of dendritic cells pulsed with HER2/neu; preliminary results have shown that the vaccine induced an intense and prolonged immune response that enables the reduction or elimination of HER2/neu expression in all treated patients. This vaccine concluded the clinical phase I, and was proposed as a new strategy in the treatment of early breast cancer and the prevention of local, regional, and distal recurrence [55,56] (Figure 1A).

#### 3.2.2. DNA Vaccines

Early studies show that cells can express genes codified into plasmids that, after being injected intramuscularly, can induce cellular and humoral immune responses against this specific antigen. Those observations are the basis for developing DNA vaccines against several diseases, including cancer [57]. DNA vaccines consist of the direct injection of DNA using plasmids that encode for antigens driven by efficient eukaryotic promoters, which could be presented by the MHC I and II to the CD4+ and CD8+ T cells [58]. After the genetic material is in the nucleus, the molecular machinery of the host will promote the transient expression of the gene of interest to obtain antigenic peptides capable of inducing immune responses [59] (Figure 1B).

In BC, MVA-BN^®^-HER2 is a recombinant vaccine vector based on the vaccinia virus; the vector encodes for a modified form of the human epidermal growth factor receptor 2 (EGFR-2). This vaccine has been evaluated as a new type of immunotherapy designed specifically for treating BC with positive expression of HER2 [60]. Complementary analysis in CT26-HER-2 animal models provided evidence about the induction of protective immune response associated with the infiltration of CD8+ T cells and the production of high levels of interferon-gamma (IFN-γ) and tumor necrosis factor-alpha (TNF-α). This vaccine is currently in clinical phase I and could be used in combination with checkpoint inhibitors (CTLA-4) to increase the overall survival in the studied model [61].

In addition to the use of recombinant vectors, other types of designs for DNA vaccines have been proposed, such as the use of cDNA. Using this design, a vaccine against mammaglobin-α was produced, which was evaluated in a transgenic mouse model with HLA-A2+. Results showed that the formulation could induce cytotoxic activity against three positive cell lines derived from BC with positive expression of mammaglobin-α (AAU-565, HBL-100 y UACC-812) that promoted the in vivo regression of established tumors [62]. Another example is WOKVAC, a DNA vaccine based on using a plasmid that encodes for epitopes derived from IGFBP2, HER2, and IGF1R, overexpressed proteins in preinvasive mammary lesions associated with progression to invasive breast cancer. These proteins are immunogenic in patients with BC and activate the humoral immune response [63].

Another example of a DNA vaccine designed for BC is SynCon FAP, the main target of which is the protein fibroblast activation protein alpha (FAPα). This vaccine showed a solid antitumor activity in different tumor models (TC-1, Brpkp110, and TSA) [64]. This specific therapeutic target has been optimized using FAPα in combination with survivin to design a new formulation of a DNA vaccine with a double target that promotes the infiltration of T cells to the tumor, thus regulating the tumor microenvironment. Also, the combination of this vaccine with Doxorubicin (Dox) promoted the antitumor effect by inhibiting metastasis, which promoted an extension of the overall survival rate in the murine model [44,65].

#### 3.2.3. RNA Vaccines

In recent years, RNA vaccines have gained attention due to their encouraging results in clinical phases [22]; these types of vaccines are based on the administration of mRNA directly to the host cytoplasm for the in vivo translation of the target antigen [66,67]. The mRNA vaccines induce robust immune responses that include the production of cytokines, such as interleukin-12 (IL-12) and TNF at the site of injection, which allows the development of an effective adaptive response against the codified antigen [68] (Figure 1C). Liu et al., 2018, used the adjuvant system of lipid/calcium/phosphate (LCP) nanoparticles (NPs) loaded with the mRNA encoding for the antigen MUC-1 and immunized Balb/c mice. They demonstrated that the mRNA encapsulated could be expressed in the lymph nodes and induce a potent and specific cytotoxic T cell response against triple-negative breast cancer [46].

Finally, using a non-coding RNA to identify the miRNA-5119 associated with regulating the expression of PD-L1. Zhang et al. evaluated the levels of ligands of immune cell inhibitory receptors and miR-5119 in dendritic cells derived from mice with BC. They found that the vaccine administration decreased T-cell depletion and suppressed tumor growth. Thus, providing evidence about the effect of the miRNA-5119 vaccine in the regulation of inhibitory receptors that enhance the antitumor immune response [69].

## 4. Peptide-Based Vaccines for Breast Cancer

Vaccines based on the use of peptides are designed for the induction of humoral and cellular immune responses. Therefore, it is essential to evaluate whether the selected peptide is capable of inducing the activation of T cells (CD4+ and CD8+) and B cells [44] (Figure 1D). The technologies used for developing these types of vaccines are safe and cheap if they are compared with traditional methods for vaccine production. Also, their high purity level avoids the presence of unnecessary compounds that could promote adverse reactions in the host [70]. A pilot study using 12 patients with mixed stages of breast cancer evaluated the effect of a peptide-based vaccine formulated using 9 peptides derived from the antigens: MAGE A1, A3, and A10, CEA, NY-ESO-1, and HER2 in combination with an antagonist of TLR3 (Toll-like receptor 3) named poly-ICLC and one peptide derived from the tetanus toxoid. The treatment was well tolerated; however, the ELISPOT assays (Enzyme-Linked ImmunoSpot Assay) could not detect any type of immune response and the vaccine did not progress to the next clinical phase [71]. Rosembaun et al., in 2020, studied a group of 7 patients with metastatic breast cancer negative to the expression of HER2 that were administered with a vaccine designed with the tumor-associated carbohydrate antigen (Tn), the toxin-derived peptide of tetanus TT_830–844,_ and the immunostimulant GSK AS15. The first results demonstrated a high production of IL-2 and antibodies IgM and IgG with the capacity to recognize and destroy positive cells to the expression of Tn by the induction of cytotoxic mechanisms associated with activating the classical pathway of the complement system [72].

There are reports about peptide-based vaccines in clinical phase II. The first is the vaccine that has as a target the folate alpha receptor (FRα) based on the use of multiple epitopes (FR30, FR56, FR76, FR113, and FR238) combined with the GM-CSF (granulocyte-macrophage colony-stimulating factor). The vaccine was capable of inducing antibody production against one or more peptides with low to moderate toxicity reactions [73]. In a different study, a vaccine that used 19 peptides derived from 11 tumor-associated antigens, such as squamous cell carcinoma antigen (3SART3), leukocyte-specific protein tyrosine kinase (Lck), prostate-specific antigen (PSA), prostatic acid phosphatase (PAP), and epidermal growth factor receptor (EGFR), was evaluated in triple-negative breast cancer patients. The presence of IgG against at least one peptide in 9 out of 10 patients was detected. In addition, a positive response of cytotoxic T cells in at least 5 of every 10 patients was found. Clinical phase II will be performed in a bigger group to establish the relationship between these results and the potential therapeutic utility [74].

A clinical phase I/II assay evaluated a peptide-based vaccine that contained peptides derived from the TAA: MUC1 (SAPDNRPAL), CEA (YLSGADLNL), and ErbB2 (KIFGSLAFL) in patients with ovarian and breast cancer. Results showed that some patients presented a positive CD8+ T cell response with IFN-γ production to at least one antigen without toxicity [75]. P10s-PADRE is a phase I/II vaccine based on using peptide mimotopes and Montanide™ ISA 51 VG as an adjuvant. This vaccine could be used in combination with standard chemotherapy in patients with triple-negative breast cancer patients. High antibody response was achieved with only three immunizations without adverse reactions [76]. Using HER2 as a therapeutic target, a novel vaccine containing the peptide GP2 (IISAVVGIL_654–662_) combined with the granulocyte-macrophage colony-stimulating factor (GM-CSF) was developed. During clinical phase I, the GP2+GM-CSF vaccine was administered in patients with BC and provided evidence about the induction of immune response and its safety [77]. Phase II concluded that patients with tumors positive for HER2 expression have a better response in combination with trastuzumab, and toxicity reactions were associated with the immunoadjuvant GM-CSF [78].

The only peptide-based vaccine in clinical phase III is NeuVax™; the efficacy of this vaccine has been tested in disease-free patients with positive and negative nodes. The formulation is based on combining the peptide E75 with GM-CSF as an immunoadjuvant [79]. Results have shown that the administration of this vaccine is safe and effective for the stimulation of in vivo immune response with the capacity to reduce the recurrence rate up to a 50% in patients with high-risk breast cancer. It has been proposed that this vaccine could be used in around 76% of the population. However, other vaccines with target peptides derived from HER2 or new targets are still under evaluation [78,80] (Table 1).

### 4.1. Immune Response after Vaccine Administration

After peptide-based vaccine administration, released peptides are captured by antigen-presenting cells (APC) by phagocytosis or endocytosis, processed, and coupled to MHC I and II molecules. Antigen presentation is performed to CD4+ and CD8+ T cells by the interaction of the TCR with MHC class I and II and costimulatory molecules, such as CD28 and CD80. CD4+ T cells could be differentiated in the subgroup Th1 and Th2. Th1 cells release IL-12 and IFN-γ, which will increase the levels of MHC molecules and promote the release of CCL3 and CCL4 chemokines for the recruitment of natural killer (NK) monocytes and cytotoxic T cells. NK cells produce perforins and granzymes to promote the apoptosis of cancer cells. CD8+ T cells could recognize the peptide derived from the TAA coupled to the MHC I in the cancer cells to promote apoptosis by releasing perforins and granzymes or by interacting with Fas/FasL. CD4+ and CD8+ T cells are capable of differentiating into effector and memory cells [86,87].

**Figure 2:**
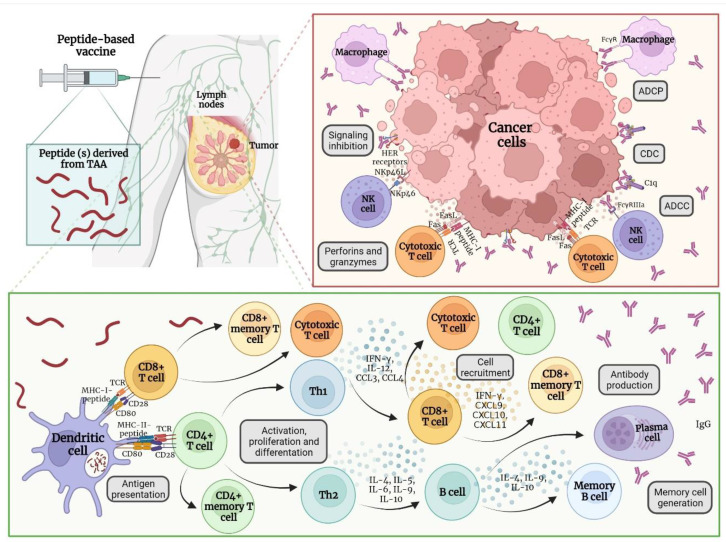
Immunological mechanism of action of the peptide-based vaccines. After its administration, the peptides will be recognized by innate immune cells that promote the activation of the adaptive immune response (cellular and humoral). Released antibodies can promote the destruction of tumoral cells by ADCC, CDC, ADCP, signaling inhibition, and complement activation (classical pathway), whereas cytotoxic T cells can promote apoptosis of cancer cells by releasing perforins and granzymes or by interacting with Fas/FasL. APC: antigen-presenting cell; MHC-I: major histocompatibility complex type I; MHC-II: major histocompatibility complex type II; Th1: T helper 1; Th2: T helper 2; ADCC: antibody-dependent cellular cytotoxicity; CDC: complement-dependent cytotoxicity; ADCP: antibody-dependent cellular phagocytosis. Image created in Biorender (https://biorender.com/ accessed on 2 July 2022).

On the other hand, the subgroup Th2 derived from CD4+ T cells will secrete IL-4, IL-5, IL-6, IL-9, and IL-10 that regulate the activation and differentiation of B-cells into memory and plasma cells that release IgM and IgG antibodies. Secreted antibodies can bind to their target TAA to inhibit the function of the target protein, thus, promoting phagocytosis through the binding to the Fc region of the antibody. Also, the activation of the classical pathway of the complement can promote the formation of the membrane attack complex (MAC). Finally, antibody-dependent cellular cytotoxicity by interacting with Fc receptors in the NK cells can promote apoptosis of cancer cells [88,89] (Figure 2).

### 4.2. New Potential Therapeutic Targets for Developing Peptide-Based Vaccines for Breast Cancer

There are several types of vaccines being developed and evaluated against breast cancer. The main differences are the type and quantity of employed antigen, adjuvant, stabilizer, or the target population. In recent years, studies about breast cancer revealed new mechanisms involved in the progression and identified multiple antigens associated with the phenotype of cancer cells. Given the potential and participation of these proteins in the development of cancer, they have been proposed as potential therapeutic targets [90,91,92].

#### 4.2.1. Syntenin-1

The melanoma differentiation-associated gene-9, also known as syntenin-1 or syndecan binding protein (SDCBP) [93], is a scaffold protein with a PDZ domain involved in the regulation of several biological processes such as cell-cell adhesion, cell-matrix, and signaling transduction. In breast cancer, this protein promotes the progression, growth, and metastasis of cancer stem cells [94]. An association between the overexpression of syntenin-1 and the tumor growth and metastasis to lymph nodes has been described in Balb/c mice [95]. It could be related to the role of this protein in controlling cellular growth during the transition from G1/S during the cellular cycle [96]. In addition to this protein being associated with multiple types of tumors, its role in cancer is notable and needs further studies.

#### 4.2.2. PLAC-1

The placenta-specific protein-1 (PLAC-1) is part of the denominated cancer/testis antigens. It is expressed mainly in the trophoblast membrane during the development of the placenta, and, in adults, this antigen is exclusively expressed in testis [97]. The report shows that the overexpression of PLAC-1 promotes cell migration and invasion in vitro and in vivo in cell lines derived from breast cancer and promotes cellular proliferation by activating the AKT pathway. These findings suggest that PLAC-1 is involved in the inflammatory response and immunological tolerance associated with immune response evasion in cancer cells [98,99]. Using in silico approaches, several epitope candidates derived from PLAC-1 have been identified for developing new formulations that, in conjunction with adjuvants, could be used in the treatment of BC [100].

#### 4.2.3. Mammaglobin-α

Mammaglobin-α (MAG) is a tumor-specific antigen of breast cancer, it is expressed in around 40–80% of all the subtypes, and it is involved in cell signaling, antitumor immune response, and chemotaxis [101,102]. It has been described that MAG possesses specificity and high immunogenicity and also regulates the tumorigenesis and aggressiveness in BC by increasing the cellular proliferation, migration, and invasion of cancer cells. MAG is responsible for activating signaling pathways such as; MAPK, FAK, MMP, and NF-κB [103], and is involved in the expression of IL-2. In vitro experiments showed that positive cells to the expression of mammaglobin could promote the activation of CD4+ and CD8+ T cells capable of recognizing and lysate cancer cells [104]. There are few studies about the potential utility of MAG as a potential therapeutic target; however, it remains a strong candidate for the diagnosis and therapy of breast cancer [105].

#### 4.2.4. NY-BR-1

The NY-BR-1 protein is a tumor-associated antigen recently described and identified in more than 70% of breast cancer tumors [106]. The expression of NY-BR-1 is more frequent in phase I carcinomas compared to phase II and III; the expression levels of NY-BR-1 are higher than HER2, and it is directly correlated with the expression of the estrogen receptor [107]. Balafoutas et al., [108] identified two peptides restricted by HLA-A2 for NY-BR-1 (p158–167 and p960–968) that could be recognized by CD8+ T cells derived from patients with BC. Due to the most frequent expression of NY-BR-1 compared with HER-2/neu (the reference target for breast cancer immunotherapy) this protein could be a valuable tool as a new therapeutic approach against BC [109].

#### 4.2.5. PRAME

PRAME is a cancer/testis antigen, mainly expressed in melanoma and other solid tumors, such as breast, lung, and kidney cancers and leukemias [110]. In BC, the expression levels of PRAME are correlated with the negative expression of ER, lower overall survival rates, and higher rates of distant metastasis [111,112]. Al-Khadairi et al., in 2019 [113] evaluated the expression of PRAME and its role in cellular migration and invasion in triple-negative breast cancer cell lines. Authors reported that PRAME promotes the aforementioned biological process through changes in the expression of epithelial-mesenchymal transition markers, such as SNAI1, TCF4, TWIST1, FOXC2, IL1RN, MMP2, SOX10, WNT11, MMP3, PDGFRB, and JAG1. In silico analysis of the chimeric protein of PRAME in combination with the adjuvant FliC-D2D3 shows that this protein could be a great candidate for developing a new vaccine against breast cancer and stimulating cellular and humoral immunity. These results increased the clinical value of PRAME as a prognostic biomarker and therapeutic target in BC [114].

#### 4.2.6. MAGE A3 and A11

The MAGE-A family (melanoma-associated antigen) has attracted much interest as cancer-associated antigens. This family contains more than 60 genes with conserved homology and similar transcriptional regulation [115]. Particularly, the expression of MAGE A3 has been reported in malignant tumors that include melanoma, brain, breast, lung, and ovarian cancer, and it is related to the repression of genes involved in antigen processing and presentation and plays an essential role in the inhibition of T cells [37]. The expression of MAGE A3 is commonly observed in more advanced stages of cancer, and it is associated with worst prognosis [116]. Another interesting member of the MAGE family is MAGE A11, which is expressed in prostate and breast cancer, and its overexpression correlates with the expression of HER-2 and ER-β [117]. Immunoinformatic approaches identified one epitope in MAGE-A11 named 9-mer: KIIDLVHLL that could be used as a potential candidate for the design of a novel therapeutic vaccine [118].

#### 4.2.7. CEACAM6

**Figure 3:**
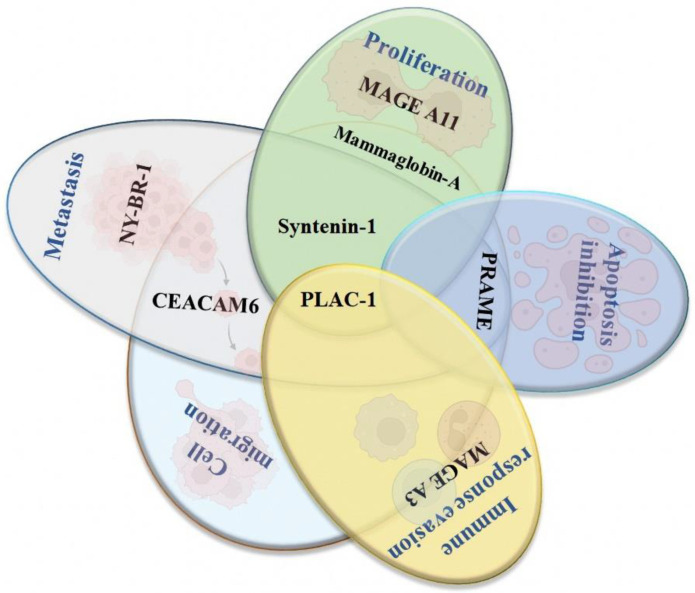
Venn diagram of novel potential targets for developing peptide-based vaccines. The selected antigens are involved in the different biological processes: proliferation, apoptosis inhibition, immune response evasion, cell migration, and metastasis. Image created in Biorender (https://biorender.com/ accessed on 2 July 2022).

Finally, the CEACAM6 (carcinoembryonic antigen-related cell adhesion molecule 6) is also overexpressed in several types of cancers, such as colorectal, pancreas, prostate, lung, and breast. Several studies have provided evidence about its role in the migration, invasion, cellular adhesion, and metastasis of cancer cells [119]. In breast cancer, CEACAM6 is overexpressed in cell lines positive to the expression of the ER; this expression was associated with the development of a more invasive and aggressive phenotype [120]. However, more evaluations need to be performed to evaluate its role in cancer and its potential utility as a therapeutic target [121] (Figure 3).

## 5. Conclusions

Immunotherapy is one of the most recurrent treatments against breast cancer and is mainly focused on the activation of the immune response and the development of immunological memory. The incidence of breast cancer keeps increasing, and there is a need to develop novel therapies that could promote the activation of the immune system as therapeutic vaccines. Different types of vaccines (whole cell, DNA, RNA, and peptide) have been proposed for breast cancer. They all have as targets TAA/TSA, which are proteins involved in metastasis, proliferation, invasion, migration, and immune response evasion. The main objective of therapeutic vaccines is the development of active immunity through the activation of cellular and humoral immune responses based in the generation of CD4+ and CD8+ T cells and B cells for antibody production that will promote immune mechanisms, such as complement, phagocytosis, NK cells, Cytotoxic T cells, and signaling inhibition.

Several peptide-based vaccines are under development, and others are currently being evaluated in clinical phases. However, this type of vaccine presents some limitations that must be overcome to accomplish the ideal therapeutic vaccine, such as the use of single peptide epitopes, immune response evasion, the failure of the candidate to elicit immune response, or the lack of efficacy during the clinical trials. Until now, the most promising vaccine has been NeuVax™ or Nelipepimut-S, which targets HER2. However, other peptide-based vaccines designed using HER2 as a single antigen or multi-epitope-based vaccines are being studied. We must take into account that multi-epitope-based vaccines are an interesting idea and could be helpful in those types of breast cancer with negative expression of HER2, to which in silico approaches emphasizing the search of immunogenic and antigenic sites in the TAA or TSA that we propose are needed.

## Figures and Tables

**Table 1:** Peptide-based vaccines derived from TAA against breast cancer.

Vaccine	Description	Clinical Phase	Evidence
NeuVax™ Peptide derived from HER2.	Breast cancer with low or moderate expression of HER2. GM-CSF and water.	III	Register: NCT01479244 [81]
GP2 Peptide derived from HER2.	Breast cancer HLA-A2+ with positive lymph nodes in tumors with positive HER2 expression.	II	[78,82]
AE37 Peptide derived from HER2.	Breast cancer with positive and negative lymph nodes, with positive expression of HER2.	II	[78]
KRM-19 Mixed vaccine of 19 peptides derived from multiple AATs.	Metastatic triple-negative breast cancer with resistance to the conventional treatment.	II	Register: UMIN000014616 [74]
Nelipepimut-S peptide + GM-CSF + trastuzumab.	Breast cancer with low expression of HER2.	II	[83]
HLA-matched personalized peptide vaccine.	Recurrent metastatic breast cancer.	II	Register: UMIN000001844 [84]
P10s-PADRE	Triple-negative breast cancer (TNBC) in stages I, II, or III.	I/II	Register: NCT02938442
Multipeptide MUC1/ErbB2/CEA	high-risk disease-free ovarian and breast cancer after completion of standard therapies.	I/II	[75]
FRα multi-epitope	Vaccine + cyclophosphamide + sargramostim in treating patients with stage II-III breast cancer.	II	Register: NCT03012100
MAG-TN3+ AS15	Breast neoplasms.	I	Register: NCT02364492 [72,85]
Mimotope P10s-PADRE/MONTANIDE ISA 51 VG	Peptide mimotope-based vaccine of tumor-associated carbohydrate antigens in patients with stage IV breast cancer.	I	Register: NCT01390064 [76]

## Data Availability

Not applicable.
